# Traffic congestion prediction based on Estimated Time of Arrival

**DOI:** 10.1371/journal.pone.0238200

**Published:** 2020-12-16

**Authors:** Noureen Zafar, Irfan Ul Haq

**Affiliations:** Pakistan Institute of Engineering and Applied Sciences, Islamabad, Pakistan; Central South University, CHINA

## Abstract

With the rapid expansion of sensor technologies and wireless network infrastructure, research and development of traffic associated applications, such as real-time traffic maps, on-demand travel route reference and traffic forecasting are gaining much more attention than ever before. In this paper, we elaborate on our traffic prediction application, which is based on traffic data collected through Google Map API. Our application is a desktop-based application that predicts traffic congestion state using Estimated Time of Arrival (ETA). In addition to ETA, the prediction system takes into account various features such as weather, time period, special conditions, holidays, etc. The label of the classifier is identified as one of the five traffic states i.e. smooth, slightly congested, congested, highly congested or blockage. The results demonstrate that the random forest classification algorithm has the highest prediction accuracy of 92 percent followed by XGBoost and KNN respectively.

## Introduction

Traffic congestion is common problem in any road network. When the number of vehicles exceeds the upper limit of the road, it causes traffic congestion having different levels of severity. Observing and supervision of traffic for real-time as well as extended-term judgment is desirable both for policy-making and the general public. The growing population in large cities causing the ever high demands of public transport has been one of the major contributing factors of traffic bottleneck problems over the years [1, 2]. Commuters are suffering longer traveling time and having a problem related to planning their journey smoothly [3, 4]. Traffic Congestion can be used for town planning [5]. Developing countries despite of having reasonable road infrastructure in their major cities are suffering from traffic congestion primarily due to their dense population. Identification and prediction of traffic congestion plays a central role in the development of Intelligent Transportation Systems. There has been interesting research on traffic congestion prediction based on real-time traffic obtained through Google Maps API [6–8]. In this paper we take Pakistan’s capital Islamabad as a case study and present traffic congestion prediction results. Islamabad and Rawalpindi are twin cities and thousands of people daily commute to Islamabad from Rawalpindi. We have employed Google Map API to extract ETA data from all the main roads of Islamabad as well as from the roads connecting Rawalpindi and Islamabad.

In this paper, our proposed system

- integrates ETA and weather data,
- labels data in accordance with ETA trends,
- identifies congestion index with time slot,
- applies machine learning techniques of Random Forest, Logistic regression and Naive Bayes, XGBoost, GradientBoost and KNN,
- and finally provides analysis of traffic patterns.

The implementation can contribute to reducing congestion and can help in free-flowing traffic. It can also help in traffic signal management. As in Islamabad, there is no availability of organized traffic datasets, this research work on Google extracted data will provide a baseline for traffic congestion prediction in Pakistan specially focused on the Islamabad area.

The rest of the paper is structured as follows. Section II discusses the related work, Section III describes the proposed solution. Section IV presents the data description and Section V describes the design and implementation of the traffic congestion map prototype. Finally Section VI concludes the paper.

## Literature review

A variety of classical machine learning algorithms and neural network models have been applied on urban traffic data to forecast congestion. Md Maksudur Rahman et al. [9] recorded the traffic blockage over days of the week and hours of the week and detected factors that cause congestion in Dhaka city. The authors investigated the influence of the number of road intersections, market places and having rickshaw free roads on the traffic intensity. They have not worked on integrated data sources. They have not applied different machine learning algorithms and have not made comparisons between peak and non-peak hours. Yuan-yuan et al. [10] have worked on online open data and have predicted traffic conditions. They used a stacked long short-term memory model. Authors used ensemble methods and improved the performance on a imbalanced dataset by infusing local trials, social media and weather information in the prediction model. The authors did not use ensemble methods and did not include weather and event data sources. Descriptive analysis of the dataset has not been performed. Muhammad Shalihin Bin Othman et al. [11] proposed a linear regression model to forecast traffic duration. For their congestion prediction, a multi-layered perceptron deep learning model is used. They have employed Weka and Google’s TensorFlow for their traffic prediction system. Some issues including unwanted loading time for forecasting congestion due to the prediction of many more clusters have bee reported. Exploratory results of the dataset are not available. The proposed MLP has only 63 percent accuracy. Google Map API has been utilized [6–8, 12] to extract data from Google’s traffic layer for traffic prediction and optimal route calculation. Viral Kapoor et al. [13] worked in a localized and distributed manner for the real-time building of informal blockage graphs over the road network and detected strong casual jamming relations. The assumptions of this paper include that all messages exchanged between taxis and RSUs are perfectly synchronous without any options of change of ordering or message loss. Accuracy is not very good and ensemble classifiers have not been tried. Yinxiang Liu et al. [14] proposed a method to predict traffic congestion based on random forest and have obtained the accuracy of 87.5 percent. They have used 1124 instances and five features such as weather, time, holiday, special condition and quality of the road. They have evaluated the model through an accuracy index. The ratio between the predicted value of the model and its actual value defined the accuracy index. The authors did not apply multiple classifiers as a comparison and statistical analysis of the dataset is not available. B. Dhivya Bharathi et al. [15] have proposed the sequential non-stationary model for predicting the bus arrival time under heterogeneous traffic conditions. They have worked on time series dataset of buses containing total 1231 trips spanning across 34 days. The performance was measured in terms of mean absolute error and mean absolute percentage error. The authors have worked on a linear model but not tried non-linear models. Nikolaos Servos et al. [16] have worked on multimodal transports with sensors equipped with transported goods. The algorithm is capable to provide congestion prediction with an even lower amount of data. They have used SVR, Extra Trees, and AdaBoost. SVR promised the best results with a mean absolute error of 16.91h. The authors did not integrate multiple data sources rather worked on only one bus service dataset. They have not predicted future route. Ning Sun et al. [17] have proposed a model to predict the traffic state of the road segments based on historical and real-time traffic information. They resolved the load balancing issue by using TPPDP LB algorithm which proposed a path with the shortest travel time to maintain global load balancing. They merged the number of parallel requests and the predicted information to maintain global load balancing. HuachunTan et al. [18] proposed the dynamic tensor completion method to find appropriate low n rank of the dynamic tensor model. The proposed Dynamic Tensor Completion (DTC) makes active use of multi mode periodic cities such as spatial information, weekly and daily periodicity, along with chronological deviations of Traffic flow. Hongjie Liu et al. [19, 20]. propose an ANN and LSTM based prediction model and suggest time feature for long distance arrival to station prediction and spatial features for short distance arrival to station prediction. Haitao Xu et al. [20, 21] proposed the dynamic road networks with the help of a time-dependent path section graph. They suggest bus arrival time prediction based on historical and real-time GPS trajectories. Time variant distributions of the travel time of path sections have been visualized through the clustering algorithm. Rafidah Md Noor et al. [22, 23] have used SVR for predicting bus arrival time. Attributes include distance of the road, peak or nonpeak hour, travel duration and weather. Weather data has not been found to play significant role in the prediction model. Xiqun (Michael) Chen et al. [24] have presented the ensemble learning approach on ride-sourcing companies such as Taxi Hailing Service, Private Car Service, Express, and Hitch. The features of the dataset are Trip travel time, trip length, trip costs, travel time, reliability of origins and waiting time fee. The authors have used boosting ensemble trees along with SVM, Naive Bayes, and Logistic Regression techniques. Boosting ensemble trees has shown the best results. Lijuan Liu et al. [25] have suggested a model which is the combination of supervised and unsupervised learning techniques. They have contained three types of features like flow features(real-time passenger flow, and previous average passenger flow and Number), Temporal features (holiday, day of a week and hour of a day) and scenario features (inbound and outbound of tickets and cards). According to the author, the model has divided into three phases. In the first phase, Temporal and scenario features are passed to the stacked autoencoders (SAE). Then pre-trained SAE passes to the supervised DNN as an input with flow features as output. In the third phase they perform prediction of the passenger flow. The hybrid approach SAE-DNN has provided promising results. Jiaqiu Wang et al. [26] summarized a Space-Time Delay NeuralNetwork(STDNN) model that works on the autocorrelation of the road traffic network locally and dynamically. STDNN model is based on three phases, namely as specification, training and implementation. Phase one involves setting up the initialized parameters and building the structure of the model. The second phase involves the optimization of parameters and the third phase involves the prediction of arrival time. They have used a dataset that contains 1200 road links with an interval of 5 minutes. An arrival and departure time information as a feature has been collected from Transport for London(TfL). STDNN obtained the best results as compare to STARIMA, Naïve and ARIMA models. Andrew Mondschein et al. [27] proposed the spatial relationships between traffic congestion and accessibility at regional and sub-regional scales. The authors have utilized a data set namely as the Southern California Association of Governments(SCAG). The authors identify how activity participation fluctuates across individuals and space in case of congestion. They have applied multivariate regression models on the dataset.traffic flows are predictable separately for the afternoon/evening weekday peaks, evenings, morning weekday, midday and weekends. The authors used a measure of activity density to measure and map household and traffic congestion trends in space. Avigdor Gal et al. [28] have proposed a model that combines both Queueing Theory and Machine Learning techniques. The authors define the natural segmentation of the data according to intermediate stops. The dataset splits in two ways firstly builds upon an extensive training set and a test set that consists of single-segment trips and secondly whole trips with the partial training set, and a long test set. Queueing Theory is used for segmentation and outlier detection. The author analyzed and predict traffic congestion using multiple statistical and machine learning models. They predicted the dataset in multiple time slots and made a comparison of peak hour and non-peak hours. they used SVM, MLP, and RNN. This paper only predicts the traffic speed and does not predict ETA. Road conditions, weather and special events have not been considered which play a significant role in the prediction of speed. The author did not make the comparison of heterogeneous speed dataset [29]. The Author works on temporal and spatial dependences concurrently, They recommend an innovative traffic forecasting method, which is the combination of the gated recurrent unit (GRU) and graph convolutional network (GCN)and model named as temporal graph convolutional network (T-GCN) model. Gated recurrent unit is used for learning dynamic changes in traffic to capture temporal dependence and GCN used to tackle spatial dependence. T-GCN was applied on spatial-temporal traffic data. In [30] the authors work on the optimization of the kernel function to capture the non-stationary characteristics of the short-term traffic speed data. The author used the wavelet de-noising approach to remove noise and short-term irregular variations from the dataset. The author provides the novel hybrid model for forecasting the short-term traffic speed. The authors worked on short-term traffic speed data but not on long-term traffic speed data. Weather, road conditions, u-turns, and the number of lanes attributes have a direct impact on the prediction of speed but these have not been considered in this paper [31]. The author proposed the multi-step prediction model to decompose the speed into residual and periodic parts. This novel approach provides the best results when the forecasting horizon is greater than 30 min. In [32] the authors identify the factors that influence traffic congestion. They merged the weather attributes with traffic attributes. To identify the factors that have a direct impact on traffic congestion, first they created a full regression model, then cleaned attributes, and finally applied residual analysis. The proposed approach achieved 84.4 percent. The proposed approach achieved 84.4 percent which may be improved through tree family models. Working on heterogeneous dataset based on speed and different machine learning models could have been interesting [33].

## Data collection and methodology

**Fig 1:**
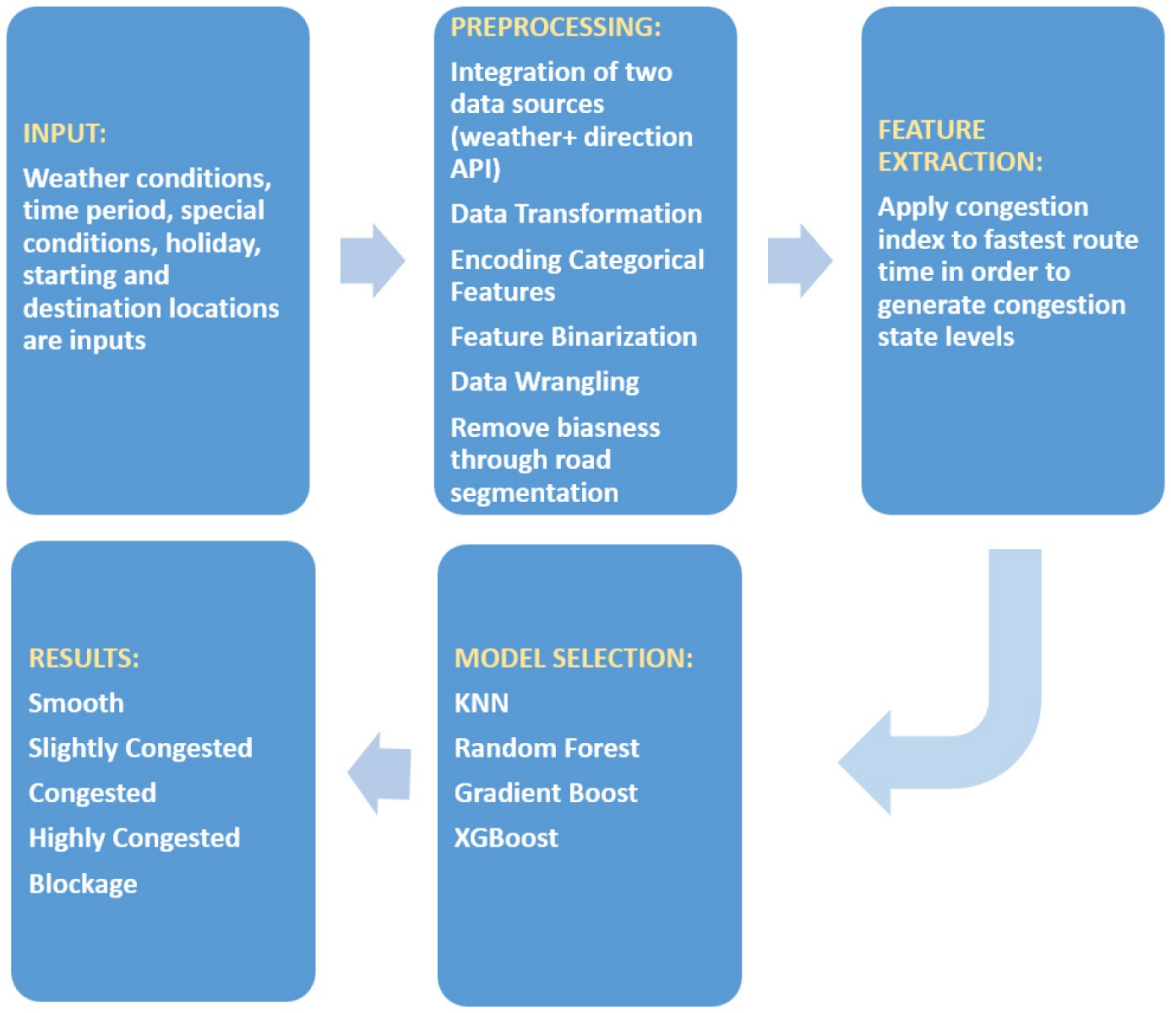
Methodology of traffic congestion prediction.

We have been collected data through Google Map API. The 123 locations cover all major roads of Islamabad as well as Murree road which is the primary road that passes through the Rawalpindi city. Rawalpindi city is a twin city of Islamabad and every day there is a huge traffic commute between the two cities. We started collecting data along the metro line that covers Murree road, stadium road, 9th avenue and Jinnah avenue but later extended to the whole road network of Islamabad. There are almost a million records that cover the traffic activity for November 2019, January 2020 and February 2020. In addition to that there are few thousand records from July 2019. The data was captured after 6-8 minutes for seven days a week and for 24 hours. Fig 1 depicts the methodology of the research. In addition to the spatial location on the map, date, time, holiday or working days, special conditions e.g. events etc and whether make up the list of features. Environmental factors affecting traffic congestion include weather conditions, different periods time, special road conditions and road quality. The extracted traffic data passes through the pre-processing phase where tasks such as data wrangling, transformation and integration are performed. Then labeling is performed to make the data suitable for supervised learning algorithms. Finally models are applied and results are obtained and discussed.

### Data description

There are mainly four types of online open data sources that deal with traffic associated data:

- traffic Maps, e.g. Google Maps and official websites of traffic management and operations.
- Social Media like Twitter, Instagram etc.
- Local events.
- Weather API.

In addition to Google Map API we extract data from open weather API.

Fig 1 depicts that the first step i.e. input consists of weather conditions, time period, special conditions and holiday along with ETA data as model input variables to establish road traffic forecasting model. Features Extraction module is used to extract information from contributions to Input module and make a gigantic dataset. Machine Learning Algorithms module is used to apply diverse machine learning algorithms e.g. Random Forest and so forth. In the Result module we check the accuracy of the Machine Learning Algorithms.

### Preprocessing

Preprocessing phase has the following sub-phases:

#### Data transformation

Data transformation makes it possible to convert the data from its given format into a particular format. This includes value transformations or normalizing numeric values to follow the min and max values. We have transformed the location in to numeric values.

#### Data wrangling

Table 1 depicts the description of attributes. Sometimes mentioned to as data munging, is the process of transforming and plotting data from one “raw” data form into another format. This may include further munging, data visualization, data aggregation, training a statistical model, as well as many other potential uses. We converted the days from string to numeric like Sunday to Saturday (0 to 6). The date is defined as day, month, and year.

**Table 1:** Data description.

Nature of Attributes	The Value of Attributes
*Day*	Monday to Sunday
*Weather*	Sunny, cloudy, rainy, mostly sunny
*Time*	Peakhour, nonpeakhour
*Holiday*	Yes, no
*SpecialCondition*	Yes, no
*Location*	112 nodes and nine segments
*Date*	Varchar

#### Encoding categorical features

Often features are not given as continuous values but categorical. For example, special condition, Time and holiday have features [“Yes”, “NO”], [“Peak_hour”, “non_peak_hour”] and [“Yes”, “NO”]. Such features can be efficiently coded as integers, for instance special condition [“Yes”, “NO”] could be expressed as [1, 0] while Time [“Peak_hour”, “non_peak_hour”] would be [1, 0] and holiday could be expressed as[1, 0].

#### Feature binariztion

Thresholding numerical features to get Boolean values is called Feature binarization. we have converted the Congestion state level into numeric values using label_binarize. So we expressed congestion labels e.g. slightly congested, smooth, blockage, congested and highly congested as 0 to 4.

### Feature extraction

The level of congestion, as defined by the system delay, may be expressed in terms of a congestion index (Cl), which is a dimensionless quantity greater than or equal to zero. A congestion index of one means that the actual travel time is twice the free-flow travel time. It is autonomous of capacity road geometry, route length and intersection control factors that could cover real transformations between two sites. The index is given by The State of the road segment and is computed from FastestRouteTime variable by the following formula:

$$\text{CI}=\left({\text{t}}_{L}-t_{O}\right)/t_{O}$$

Where,

t_*L*_ = *the current time for the road segment*;

t_*O*_ = *the least time for the road segment*.

Table 2 enlists various CI ranges and their corresponding labels from smooth to blockage. As different segments of the road network have varying number of lanes, smoothness, traffic volume etc, they need to be labeled separately based on their own Congestion Index calculations. The following algorithm takes into account different road segments composing together into the road network under discussion, calculates their Congestion Indices and applies congestion labels accordingly.

**Table 2:** Congestion state level.

*CI*	Traffic State Level
(0, 0.15)	Smooth
(0.15, 0.35)	Slightly Congested
(0.35, 0.65)	Congested
(0.65, 2.0)	Highly Congested
*Above* 2.0	Blockage

**Fig 2:**
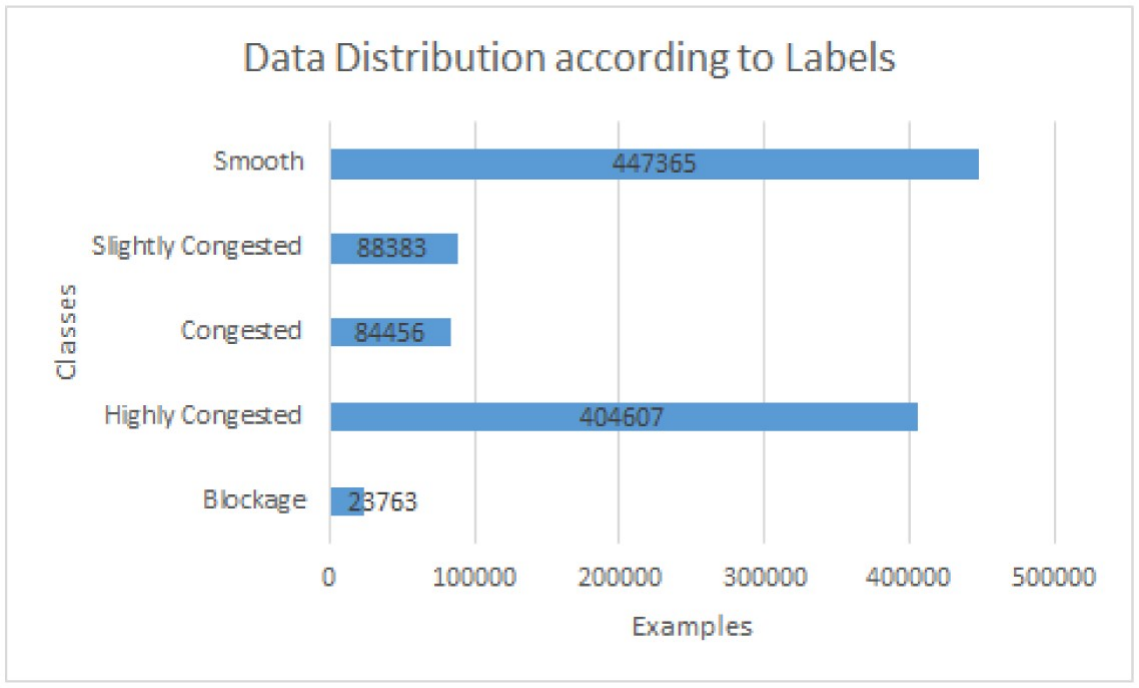
Data distribution according to labels.

Fig 2 depicts the data distribution according to labels. It is interesting to note that there are large number of highly congested examples. As the data was collected 24 hours from the roads therefore the volume of smooth traffic is also affected by data retrieved during night hours.

**Fig 3:**
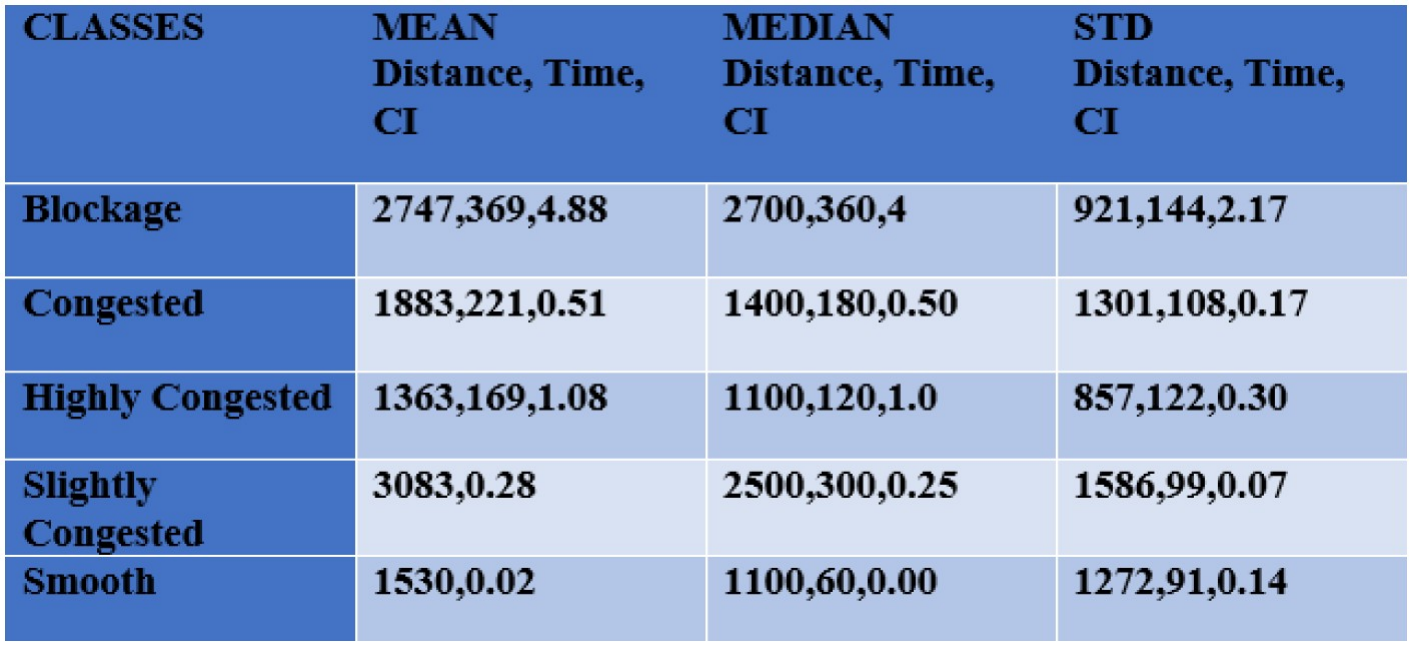
Descriptive analysis.

Fig 3 depicts the statistical description of the dataset. We have calculated the mean, median, and std of the label class. Road segments have different lengths. Congestion Index also helps to normalize the traffic activity on road segments of varying length.

**Algorithm 1**: Segmentation normalization of *CI*

*Segmentize Distribution of records*;

*Label records of each segment*;

**foreach** *Segment* *S_i_* **do**

 t_*o*_ = *min*(*ET* *AinS*_*i*_)

 **while** *i* < *len*(*SegmentS*_*i*_) **do**

  Compute $CI_{s}=\frac{t_{i}-t_{o}}{t_{o}};$  *height 0.4pt*

  apply label on the record containing t_*i*_

 **return** SUCCESS;

## Descriptive and exploratory analysis

**Fig 4:**
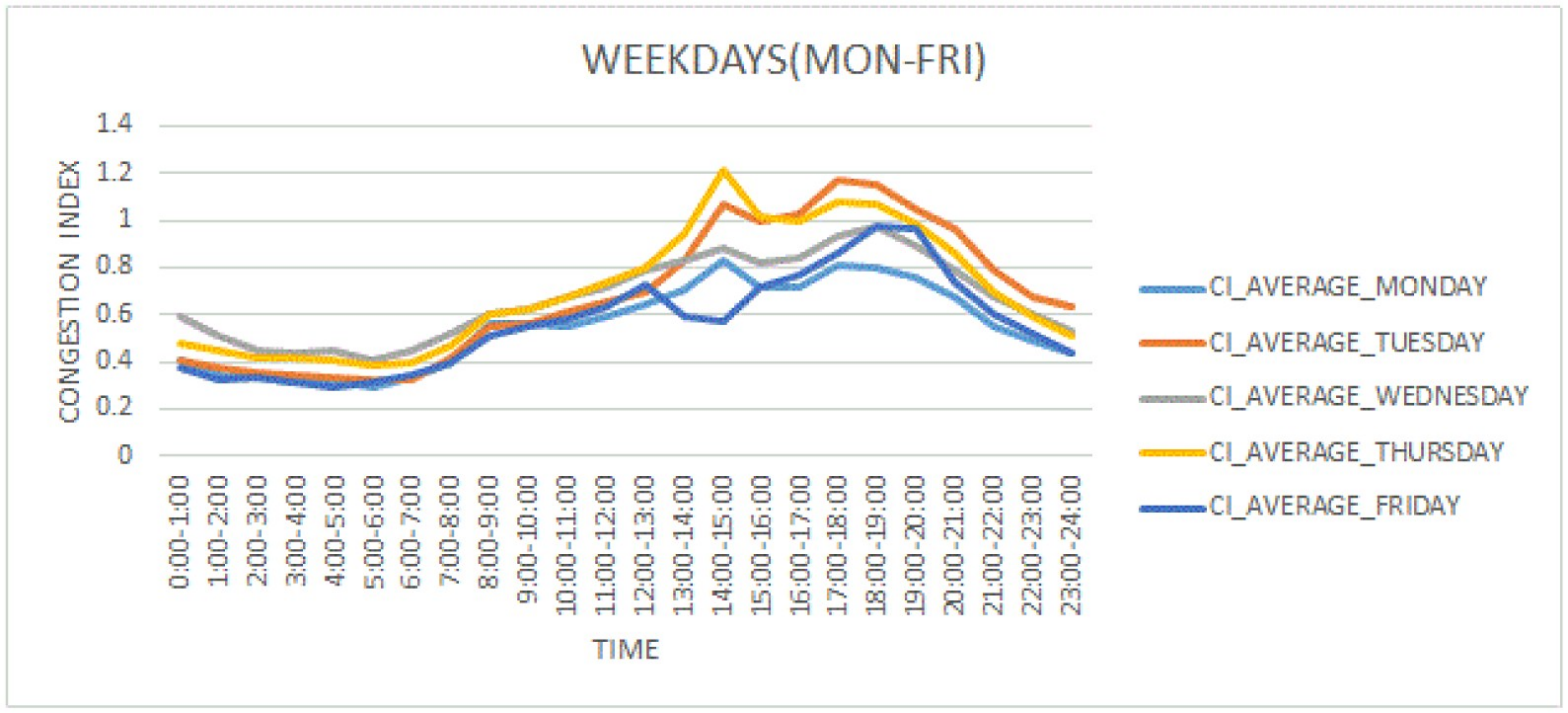
Congestion index variations on weekdays.

Fig 3 provides the mean, median and standard deviation of all five labels of data. The distance is given in meters whereas the unit of time is seconds. Congestion Index (CI) is a derived attribute, a ratio that is utilized to label classes. CI also provides a normalized perspective of the congestion as road segments are not exactly of the same size. Fig 4 presents the congestion index vs. time of day on weekdays. Different working days, i.e. Monday to Thursday are shown with different color lines. From Fig 4, it can be visualized that there is a major deviation in the congestion index over different hours of the day. In the morning rush hour (8:00-9:00 am), the congestion index is 0.6 which is higher than the average of morning hours. During rush hours (2:00-3:00 pm) the congestion index is 1.2 which is higher than all of the day time. During the evening rush hour (around 6:00—7:00 pm), the congestion index is 1.1. The behavior of the congestion index in the different time slots is significant for model development and taking the average of the congestion index for each time slot can be helpful in predicting congestion based on historical data. Time slots 8:00-9:00am, 2:00-3:00pm, and 6:00-7:00pm show highly congestion.

From Fig 4, it can be visualized that there is a major deviation in the congestion index over different hours of the day. Friday’s congestion index is quite different from different weekdays. On Friday, during the morning rush hour (8:00-9:00 am), the congestion index is 0.5 which is higher than the average of morning hours. Friday traffic is different from other weekdays due to the Friday prayer which is offered during 1-2 pm. During rush hour(12:00-1:00 pm) congestion index is 0.7 which is higher than all the time of day. During the evening rush hour (around 6:00—8:00 pm), the congestion index is 1.0.

**Fig 5:**
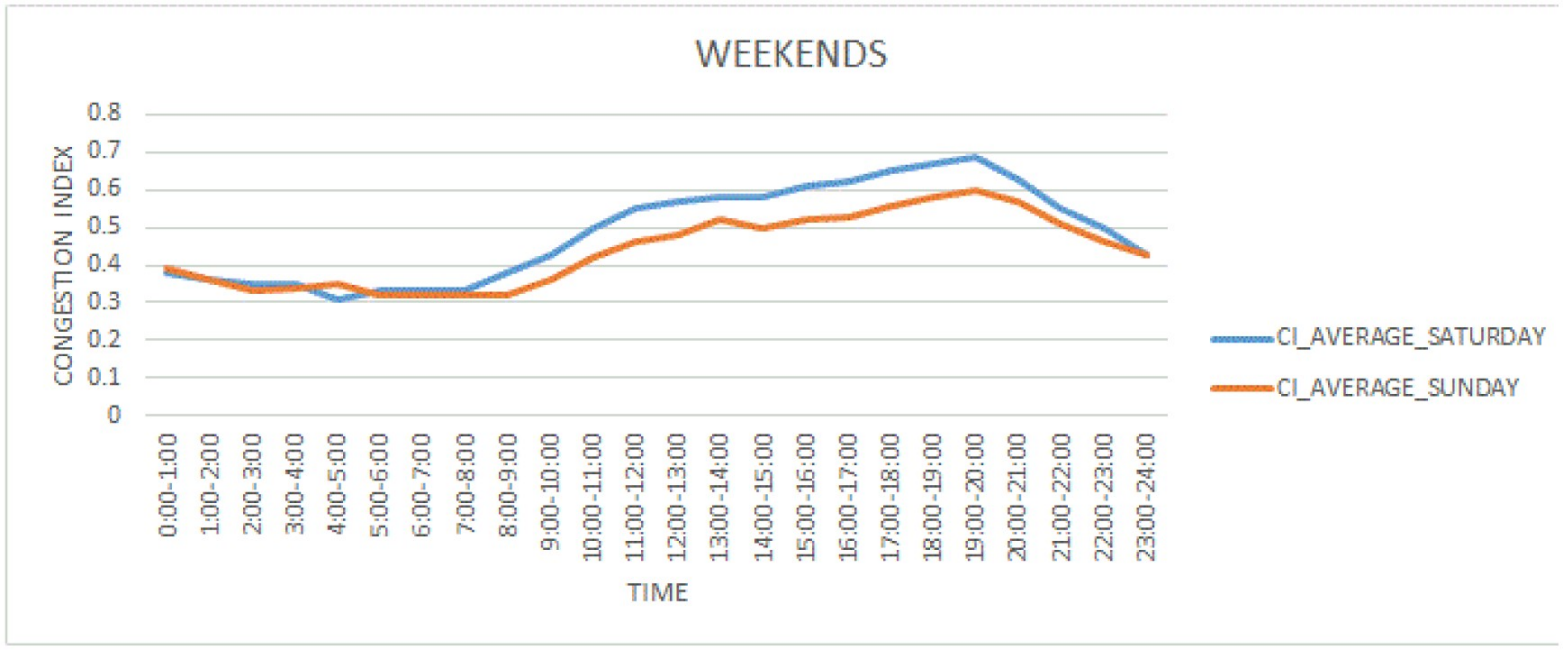
Congestion index variations on weekends.

Fig 5 depicts congestion index variations on weekends. This figure shows that weekends behavior is quite different from weekdays.

**Fig 6:**
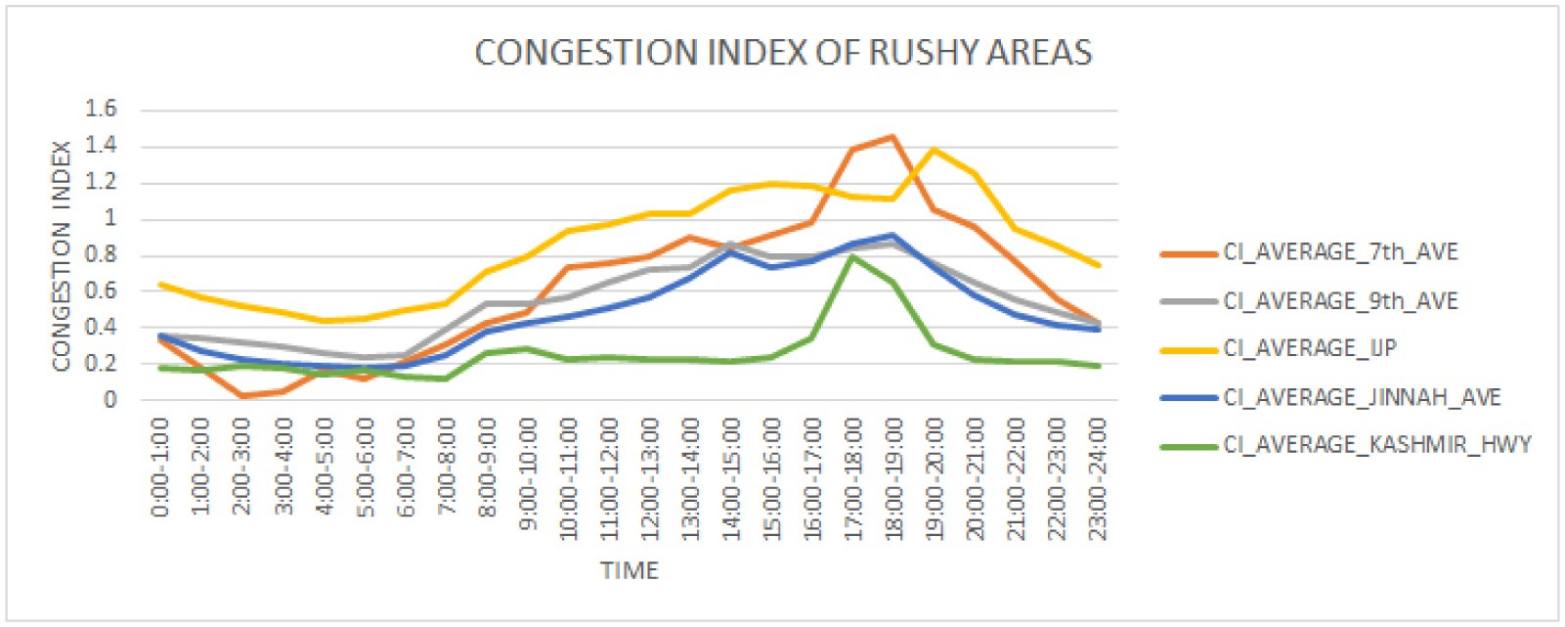
Congestion index trends in rushy areas.

Fig 6 shows distribution of traffic on different roads. 7th Ave is smooth from 9:00 to 10:00 am, congested from 10:00 am to 5 pm, and highly congested from 5:00 to 7:00 pm. The 9th Ave road is congested from 8 to 9 am, 2:00 to 3:00 pm and 6 to 7 pm. IJP road which is usually crowded by logistic trucks is highly congested from 10:00 am to 10:00 pm. Jinnah Ave which runs across the main business area is congested from 2:00 to 3:00 pm and 6:00 pm to 7:00 pm. Kashmir highway that is utilized mostly by office workers is congested from 5:00 pm to 7:00 pm.

## Model selection

The first step i.e. input is using weather conditions, period time, special conditions and holiday as model input variables to build a road traffic forecasting model. Features Extraction module is used to extract information from contributions from the Input module and make a huge dataset. Machine Learning Algorithms module is used to apply diverse machine learning algorithms e.g. Random Forest, XG boost, gradient boost, KNN SVM and so forth. We have applied different machine learning algorithms and tuned their respective parameters for optimized results. We found out that Tree based algorithms perform better than others. Therefore we have fine tuned our results on various tree based algorithms. The following decision tree based algorithms gave promising results:

### Random forest

Random forests or random decision forests are an ensemble learning method for classification, regression and other tasks that operates by constructing a multitude of decision trees at training time and outputting the class that is the mode of the classes (classification) or mean prediction (regression) of the individual trees.

The psuedocode of the Random Forest algorithm is given as follows:

1. Randomly choose “L” distinctive attribute from total “n” attributes.
2. Where L less than n.
3. Among the “L” distinctive attribute, compute the node “m” using the finest split point.
4. Divide the node into daughter nodes using the best split.
5. Recap 1 to 3 steps until “l” number of nodes has been reached.
6. Construct forest by iterating steps 1 to 4 for “k” number times to create “k” number of trees.

### XGBoost

In boosting, the trees are constructed sequentially such that each successive tree objectives to shrink the errors of the previous trees. Each tree updates the residual errors and learns from its predecessors. Hence, the tree that develops next in the structure will learn from a reorganized version of the predecessors. XGBoost (eXtreme Gradient Boosting) is an advanced implementation of a gradient boosting algorithm. It is a perfect combination of software and hardware optimization techniques to yield superior results using less computing resources in the shortest amount of time. XGBoost is used to handle missing values and provide regularization to avoid overfitting and bias and provides built-in cross-validation.

### Gradient Boost

It is a special case of boosting where errors are minimized by a gradient descent algorithm. It is used to minimized errors in the sequential model.

## Results and discussions

The dataset consists of 9 attributes named as Day (day of the week), System Time (current time), Weather (weather conditions), Time (peak hours/non-peak hours), Holiday (yes/no), Special Conditions (refer to any condition which can cause an increase in traffic e.g. accidents), Starting Location, Destination Location and Fastest_Route_Name. It consists of 1 target variable named as State (congestion state of the road segment i.e. Smooth, slightly congested, Congested, highly congested or Blockage). The dataset is composed of 1048576 records and consists of nine segments. Day and System Time attributes are collected directly from the system. Weather is collected through OpenWeatherMap API and Time, Holiday, Special_Conditions are collected manually from the user of the server after specified intervals.

Traffic data is retrieved from Google Maps API by which Starting_Location, Destination_Location, Fastest_Route_Name and Fastest_Route_Time (temporary variable) are formed from the response (JSON) of the Google Map API from 123 collection points.

Table 3 depicts that tree family yields the most promising results and SVM does not produce any good results.

**Table 3:** Accuracy of different models.

Model Name	Accuracy
*RandomForest*	92 percent
*XGBoost*	91 percent
*KNN*	91 percent
*GradientBoost*	83 percent
*OnevsRestClassifier*	61 percent
*LogisticRegression*	50 percent
*Na*ï*veBayes*	47 percent
*SVM*	39 percent

The evaluation metric used is K-folds cross-validation, which divides the data into folds and then trains the data for total minus 1 folds and predicts for the untrained fold and calculates its accuracy score. The process is repeated until every fold gets a chance to be evaluated. Finally, the accuracy score for every fold is averaged at the end to calculate the cross-validated score. In this implementation 10 folds are used to evaluate models. The cross-validated scores of multiple algorithms are shown through bar char.

**Fig 7:**
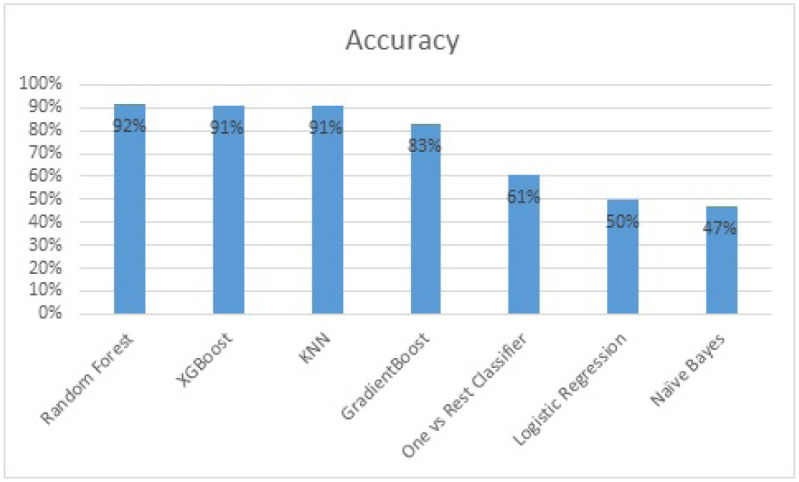
Cross-validated scores of multiple algorithms.

**Fig 8:**
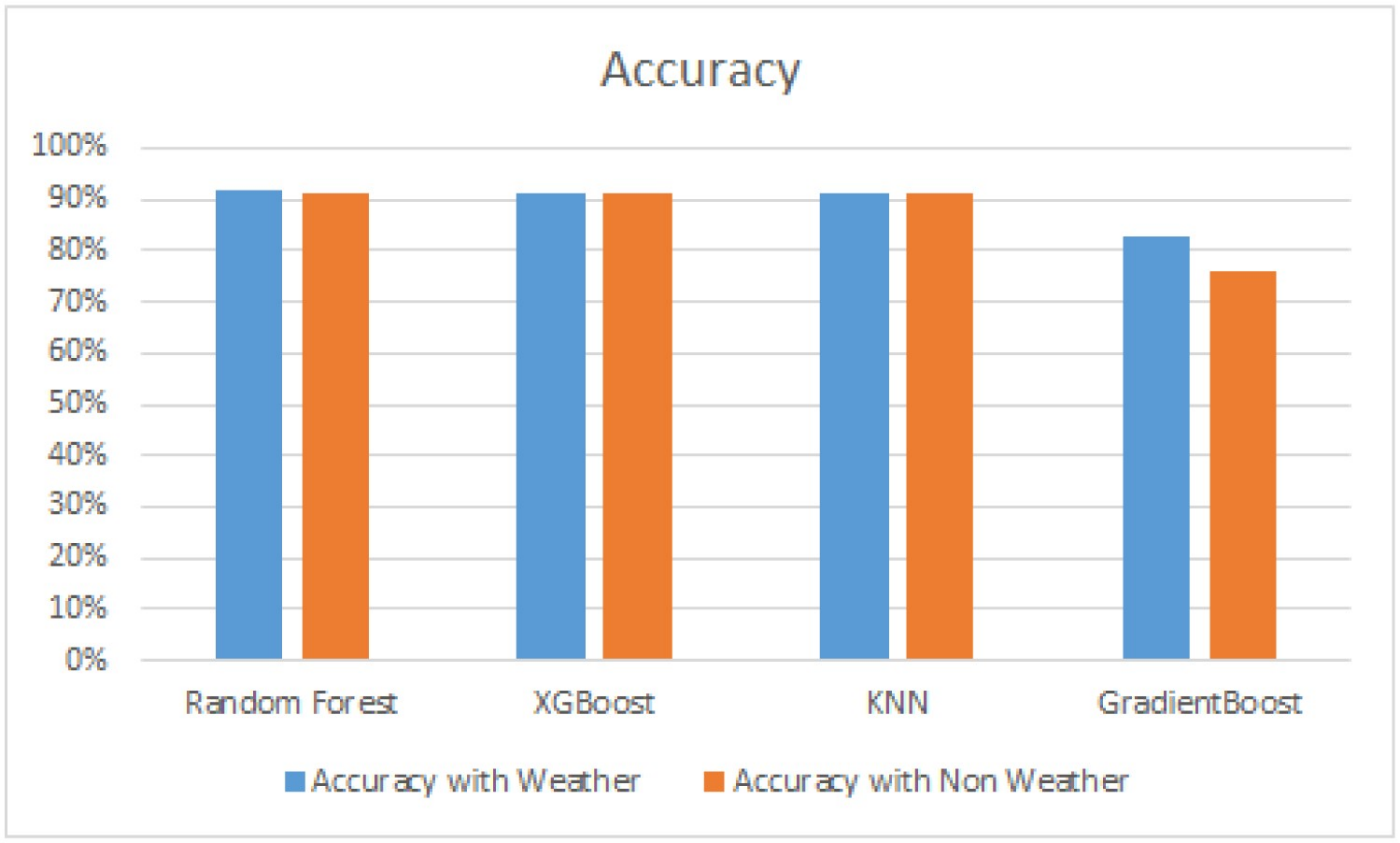
Cross-validated scores of multiple algorithms with weather and non weather.

In the Fig 7 random forest is shown to yield the highest accuracy as compared to other tree family models and classical supervised learning techniques. The accuracy of different algorithms are shown below:

We have also observed that there is only a slight effect of weather conditions on the accuracy of algorithms. Islamabad does not have any severe weather such as snowfall. The impact of weather is likely to increase if more accurate data from the weather stations of the region is utilized. Fig 8 depicts the effect of weather on the accuracy of algorithms used. We have performed experiments with the integration of weather data source with ETA data source. With the integration of weather data source results are more improved specially gradient boost results are improved from 76 percent to 83 percent whereas other models slightly improved their accuracies. Random Forest could improve its accuracy from 91 percent to 92 percent only. The weather data was obtained from the OpenWeather API which provides city wise prediction and does not have high spacial resolution. It is very likely that if the weather data had been obtained from weather stations deployed in the vicinity of the roads, the results would have been much better.

**Fig 9:**
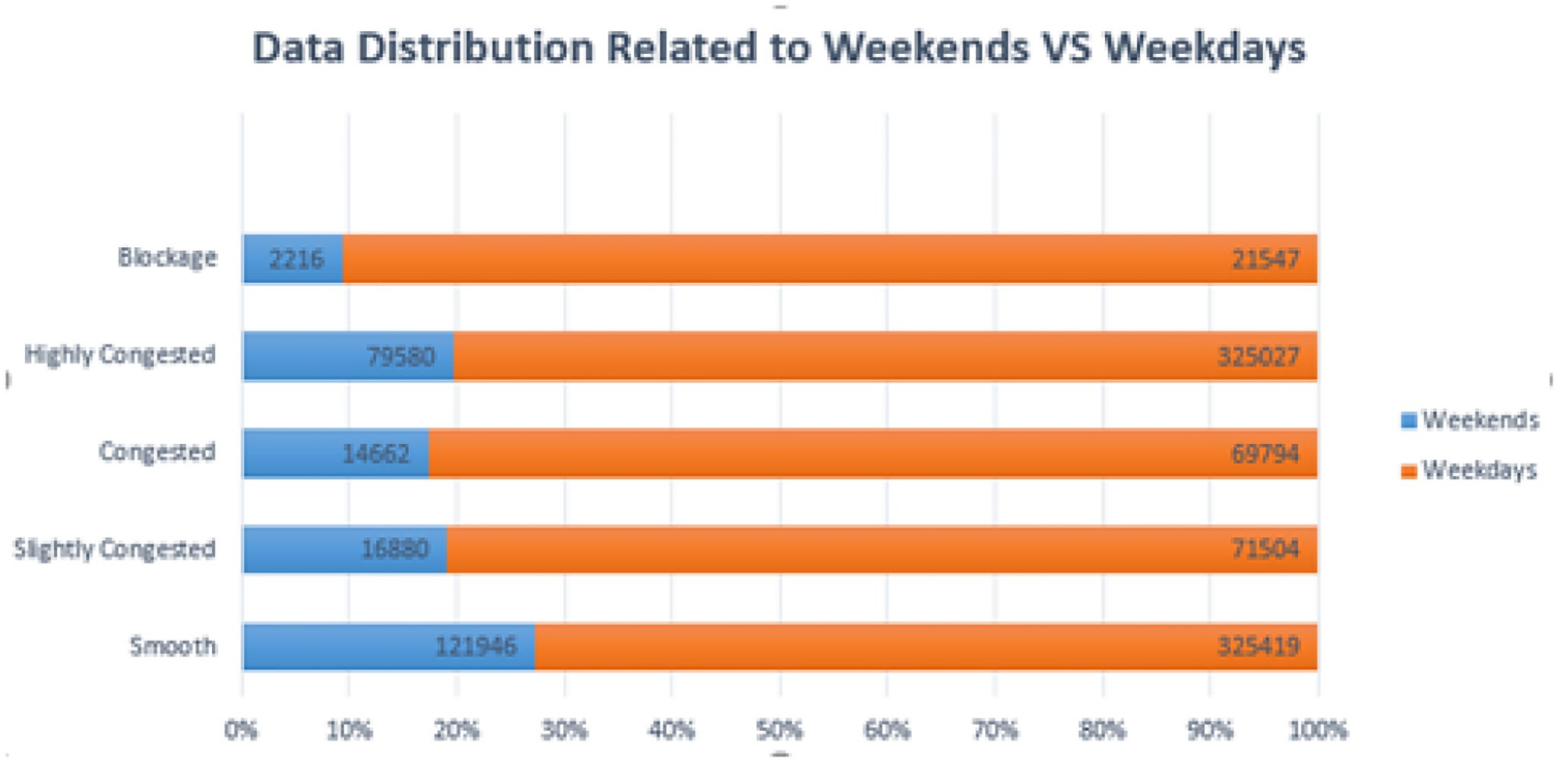
Data distribution according to weekends and weekdays.

Fig 9 depicts the labels distributed across weekdays and weekends. We have a total of 1048575 instances in which 235284 instances are weekends and 813291 are weekdays. In weekends 121946 examples are related to smooth class, 16880 examples belong to slightly congested, 14662 are congested, 79580 are highly congested and 2216 belongs to blockage examples. On the other side, 325419 belongs to smooth, 71504 are slightly congested, 69794 congested, 325027 highly congested and 21547 belongs to blockage class on weekdays.

**Fig 10:**
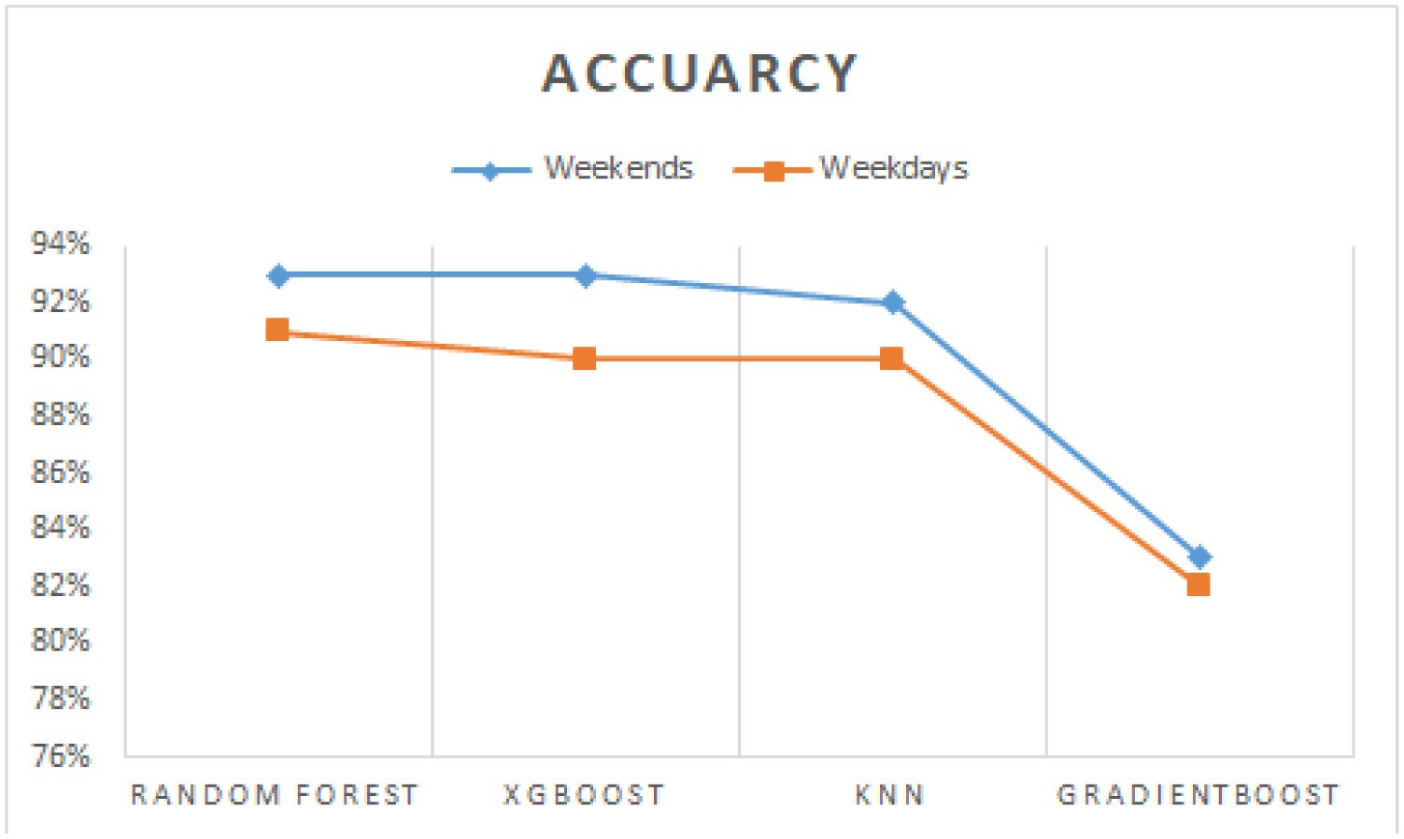
Cross-validated scores of multiple algorithms with weekends and weekdays.

We have performed experiments to make a comparison between weekends and weekdays. Results depicted in Fig 10 reflect that models give better accuracy at weekends as compared to weekdays. It is interesting to note that Islamabad being the capital of Pakistan draws a lot of traffic across other cities specially on Friday afternoon and on Sundays as there are a large number of persons whose workplace is in Islamabad.

**Fig 11:**
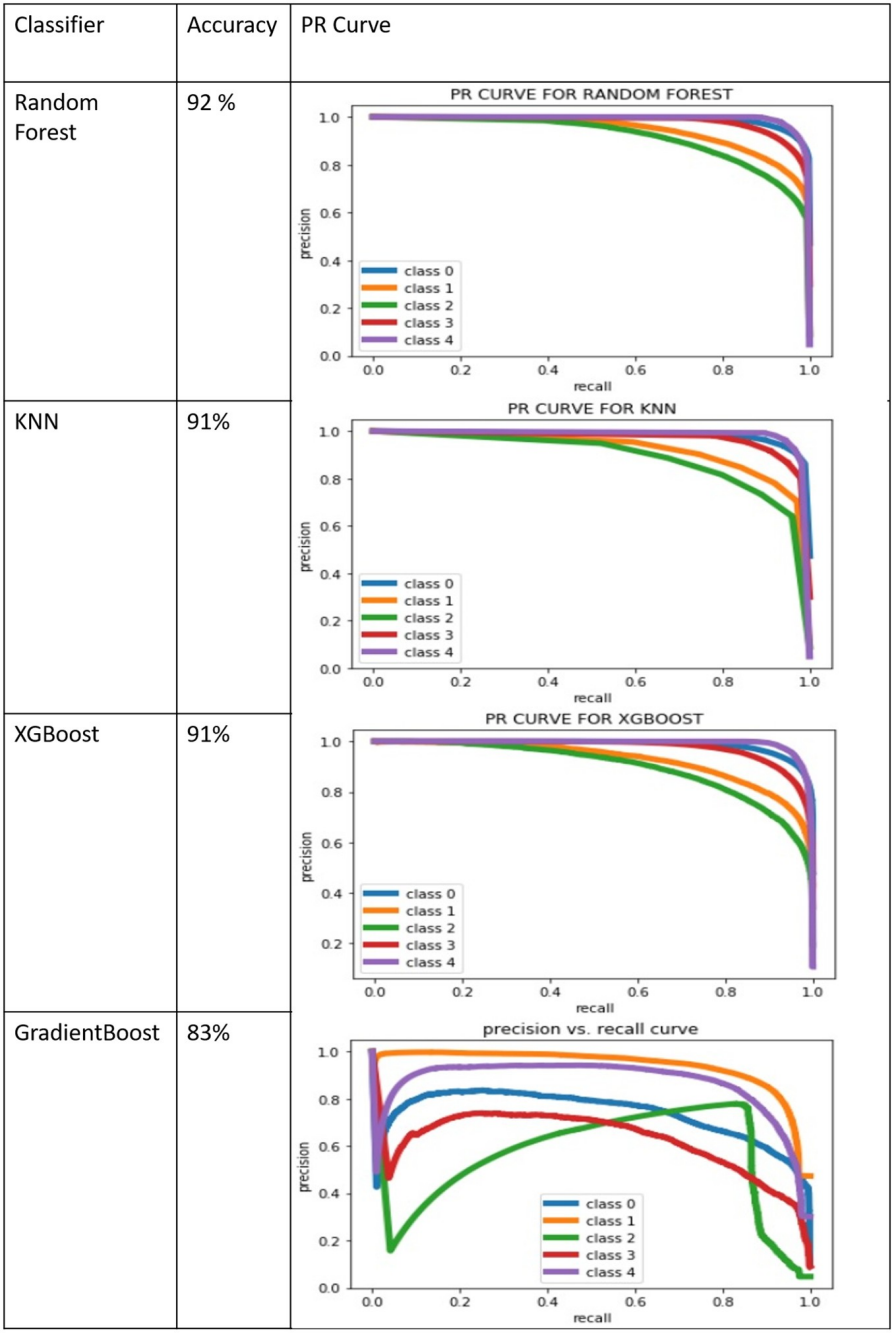
PR Curve for best scoring classifiers.

As our dataset is imbalance, we have drawn PR Curves for the analysis of performance. PR Curves of the output for the best scoring algorithms are shown in the Fig 11. In this PR Curve Class 0 means smooth, Class 1 means slightly congested, Class 2 means congested, Class 3 means highly Congested and Class 4 means blockage.

Random forest gives the best accuracy i.e 92 percent among many classifiers. In case of Random Forest the PR curve for the classes representing smooth and blockage are almost perfect approaching to 1. This indicates exceptionally high accuracy.

The detailed breakdown of the best performing algorithm Random Forest in terms of precision, recall, F1 score and support is given in Table 4. KNN yields similar results with accuracy 91 percent. The overall performance of KNN is almost identical to that of Random Forest. XGBoost also delivers good results but it takes much more time for training as compared to Random Forest.

**Table 4:** Accuracy report for random forest.

Precision	Recall	F1-Score	Support	Class
0.93935	0.94671	0.94301	189026	smooth
0.85013	0.86480	0.85740	37597	slightly congested
0.82933	0.81182	0.82048	35578	congested
0.92218	0.91446	0.91831	120020	highly congested
0.96441	0.94512	0.95467	18751	blockage

Gradient Boost algorithm does not very well in this case as compared to other tree based algorithms and is able to deliver only 83 percent accuracy. In case of Gradient Boost does not possess capability to optimize performance parameters dynamically. XGBoost, which is an advanced version of the Gradient Boost on the other hand can optimize performance parameters dynamically on its own.

Table 5 shows results of XGBoost after parameter tuning. The hyper parameter of the XGBoost includes objectives, number of estimators, learning rate and maximum depth. The XGBoost performs best with low n estimators and low depth. The loss function Binary:logistic seems to perform better than others when combined with max depth 2, learning rate 1 and n estimators 40. Table 6 enlists Gradient Boost results after parameter tuning. The maximum accuracy is 83 percent which is a combination of learning rate equal to 1, max depth 2 and max features 9.

**Table 5:** XG Boost results after parameters tuning.

Objective	n estimators	Learning rate	Max depth	Accuracy
*Binary* : *logistic*	40	01	2	91percent
*Binary* : *logistic*	100	0.01	4	68percent
*Binary* : *logistic*	100	1	3	86percent
*Binary* : *hinge*	100	1	3	86percent
*Multi* : *softmax*	100	1	3	52percent
*Multi* : *softprob*	100	1	3	74percent
*Count* : *poisson*	100	1	3	86percent
*Reg* : *tweedie*	100	1	3	79percent
*Reg* : *squarederror*	100	1	3	86percent

**Table 6:** Gradient Boost results after parameters tuning.

Learning rate	Max depth	Max features	Accuracy
0.05	2	2	52percent
0.75	2	2	72percent
1	2	2	74percent
0.75	2	9	75percent
1	2	9	83percent
1	3	9	63percent

**Fig 12:**
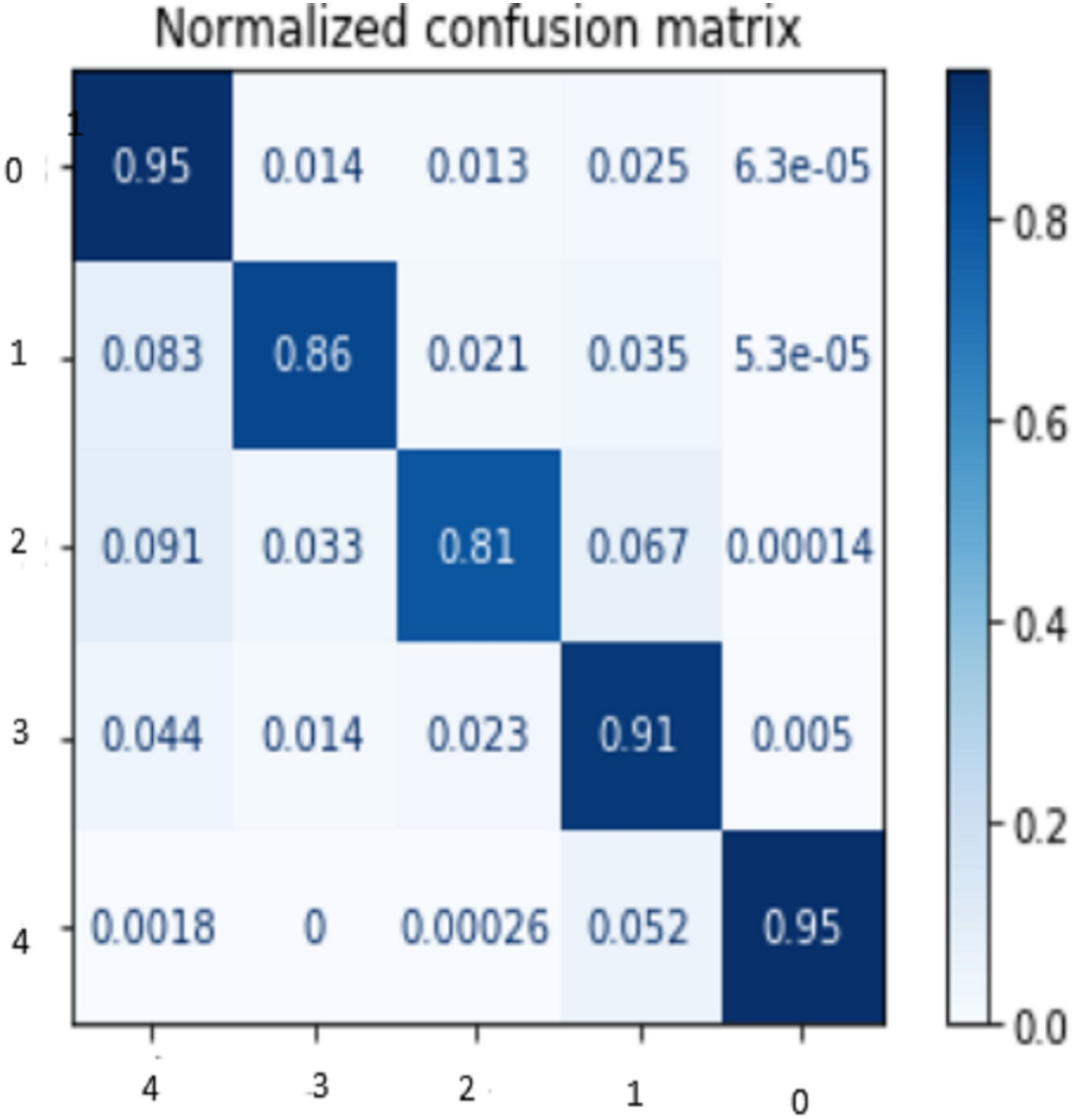
Confusion matrix for random forest.

Fig 12 shows the error matrix of the best performing algorithm i.e. random forest. In this Figure diagonal shows the True Positive elements. Class 0 which represents smooth traffic shows 0.95 true positive elements where as class 1 that represent slightly congested has 0.86, class 2 that depicts congested has 0.81, class 3 that represents congested has 0.91 and class 4 that stands for blocked has 0.95 true positive elements. Thus means smooth and blockage show the highest accuracy. We have opted to present the confusion matrix of the best performing algorithm. The second best performing algorithm XGBoost yields similar results.

Table 5 depicts how XG Boost dynamically optimizes parameters. Asit employs one vs all approach, we observe maxdepth 2 produces the most promising results. XG Boost contains hyper-parameters such as objective which is the loss function, n estimators, learning rate, and Maxdepth. In XG Boost, objective Binary: Logistic, n estimators = 40, learning rate 01, max depth 2 gives 91 percent accuracy.

In Table 6, Gradient Boost contains hyperparameters such as learning rate, max depth, and max features. In Gradient Boost hyperparameters learning rate 01, max depth = 3, and max features = 9 provide an accuracy of 83 percent. We choose max depth 3 because the dataset contains 9 features and as the learning rate means step size, fastest step size is 1. In the case of hyperparameter max features are nine.

In Table 7, KNN contains two hyperparameters i.e n neighbors and weights. Weight is the distance measure that is used to find the nearest neighbors. In KNN, the combination of hyperparameters with n neighbors = 05 and weights = uniform provide an accuracy of 91 percent. We choose n neighbors 5 because the dataset contains 5 labels or classes.

**Table 7:** KNN results after parameters tuning.

n neighbors	weights	Accuracy
5	uniform	91percent
5	distance	91percent

### Conclusion

Retrieving dataset from Google Maps API, this study carried out road congestion assessment and prediction of Islamabad City as a case study. This paper utilizes the ETA based congestion index as the road network state evaluation indicator and thus distributes the traffic state into five categories ranging from smooth to blockage. We integrated the traffic dataset with the weather dataset and applied different machine learning algorithms. As Decision Tree based algorithms gave the best results we further specialized into this class of algorithms. We found out that Random Forest and XG Boost provide the best resutls. In future we plan to integrate the GPS data from tracking devices with the traffic ETA data and study the results.

## Supporting information

S1 Code (RAR)

S1 File (RAR)

S1 Dataset (CSV)
